# Potency of teleophthalmology as a detection tool for diabetic retinopathy

**DOI:** 10.1038/s41598-023-46554-6

**Published:** 2023-11-10

**Authors:** Liu Li, Yu Jin, Jun Hua Wang, Sha Sha Wang, Fang Xiu Yuan

**Affiliations:** Department of Ophthalmology, Nanchang First Hospital, Jiangxi, 330038 China

**Keywords:** Endocrine system and metabolic diseases, Eye diseases, Engineering

## Abstract

In China, the prevalence of diabetic retinopathy (DR) is increasing, so it is necessary to provide convenient and effective community outreach screening programs for DR, especially in rural and remote areas. The purpose of this study was to use the results of ophthalmologists as the gold standard to evaluate the accuracy of community general practitioners' judgement and grading of DR to find a feasible and convenient DR screening method to reduce the risk of visual impairment and blindness in known diabetes patients. Retinal images of 1646 diabetic patients who underwent DR screening through teleophthalmology at Nanchang First Hospital were collected for 30 months (January 2020 to June 2022). Retinal images were collected without medication for mydriasis, stored by community general practitioner, and diagnosed by both community general practitioner and ophthalmologist of our hospital through teleophthalmology. The grading of ophthalmologist was used as a reference or gold standard for comparison with that of community general practitioner. A total of 1646 patients and 3185 eyes were examined, including 2310 eyes with DR. The evaluation by the community general practitioner had a Kappa value of 0.578, sensitivity of 80.58%, specificity of 89.94%, and accuracy of 83.38%% in 2020; a Kappa value of 0.685, sensitivity of 95.43%, specificity of 78.55%, and accuracy of 90.77% in 2021; and a Kappa value of 0.744, sensitivity of 93.99%, specificity of 88.97%, and accuracy of 92.86% in 2022. Teleophthalmology helped with large-scale screening of DR and made it possible for community general practitioner to grade images with high accuracy after appropriate training. It is possible to solve the current shortage of eye care personnel, promote the early recognition of disease and reduce the impact of diabetes-associated blindness.

## Introduction

In recent years, the number of diabetes patients has shown a rapid upwards trend and has become a global public health problem. The number of diabetes mellitus (DM) patients in China is approximately 110 million^1^. Diabetic retinopathy (DR) is a kind of retinopathy caused by diabetes patients' long-term hyperglycaemia, which leads to retinal ischaemia, hypoxia or retinal haemorrhage. The incidence rate of DR among diabetes patients is as high as 50%, and it is a common blinding disease. Therefore, timely diagnosis of DR is of great importance to improve its follow-up treatment.

The coronavirus disease 2019 (COVID-19) pandemic disrupted routine DR screening^2^. First, patients' worries about contracting COVID-19 during their stay in the hospital prevented them from coming to the hospital for treatment. During this deadly pandemic, when health care services reached their limits, retinal screening for asymptomatic patients was not a priority. There is almost no distance between the examiner and the examinee during fundus examination. The risk of close contact with patients with potential subclinical COVID-19 infection may also have led to doctors' unwillingness to perform ophthalmoscopy. In fact, ophthalmologists had a higher risk of contracting COVID-19^3^. Therefore, this encouraged us to look for an alternative to fundus screening.

Teleophthalmology might represent an opportunity to improve screening for DR in developing countries, and teleophthalmology with links to an ophthalmologist may enable regular repetitive DR screening covering a wider population. A nonmydriatic fundus camera was equipped, and by connecting to the internet, we used computers and even smartphones to remotely browse and grade the retinal images obtained. The purpose of this study was to explore the effect of the use of teleophthalmology consultations for screening for DR in diabetic patients and to determine a feasible screening scheme for this condition.

## Materials and methods

This was an observational retrospective study. The data of urban diabetes patients who underwent DR screening at Nanchang First Hospital from January 2020 to June 2022 were collected. A teleophthalmology platform was built that was organized by the internet website “Eye Grader" (with website was https://www.eyegrader.com/,hereafter referred to as the website) and the nonmydriatic fundus camera system in the hospital.

The website is a remote medical cloud platform that integrates fundus colour photography, film reading, and referral. The basic components of the Eye Grader ophthalmic remote medical integration system include collection points and film reading grading centres. The system is designed based on a cloud computing platform. The fundus photography images collected in communities and cooperative medical points are transmitted to the film reading and grading centre through the internet. Trained technical personnel complete the film reading work through internet-accessible computers or even mobile phones and generate a film reading report for feedback to the collection point. Patients receive paper reports or can directly search for them through WeChat. Patients with severe conditions are referred to the hospital according to indications, forming an integrated system that connects patients, grassroots hospitals, and tertiary ophthalmic centres. Large hospitals not only assist grassroots hospitals in diagnosing fundus diseases but also improve their service volume and regional radiation capabilities through the referral of complex cases. The Eye Grader system also allows for arbitrary addition of collection points and grading centres for film reading.

The same nondilated fundus camera, model TRC-NW300, produced by Topcon, was used for examination. Patients did not need to undergo dilated pupil treatment. After the patient had adapted to a dark environment for 5 min, a photo of one eye of the patient was first taken, and a 45° colour photo of the posterior pole of the fundus was taken with the macula as the centre. After resting for a while and waiting for the patient's pupils to recover, another photo of the eye was taken, with one image taken for each eye. Community general practitioner collected retinal images, encrypted clinical data, and stored the data on the remote consultation platform.

This study was approved by the ethics committee of Nanchang First Hospital; data were anonymized during extraction, making it impossible for patients to be identified. All experiments were performed in accordance with relevant guidelines and regulations, and patients signed an informed consent form.

The following data were recorded: age, sex, course of diabetes and whether the patient had hypertension.

DR was defined based on fundus photos by the same community general practitioner and the same expert ophthalmologist (according to the website's built-in grading system), and the diagnosis results of the ophthalmologist were the final diagnosis and classification. First,the ophthalmologist and the community general practitioner learned DR grading by themselves through Eye Grader for 1 day,and the ophthalmologist was certified DR grader then.The community general practitioner compared the grading on the website with his own grading after the ophthalmologist responded to the final grading to improve accuracy every week. Those who could not obtain fundus photos for DR diagnosis due to anophthalmos or severe refractive interstitial opacity, such as cataracts, were excluded.

The DR grades were as follows:

1. R0 = None or no DR
2. R1 = Microaneurysms (MAs), hard exudate (HE) or cotton wool spots (CWSs)
3. R2 = Venous beading (VB), duplication, multiple blot haemorrhages or intraretinal microvascular abnormalities (IRMAs)
4. R3a = New vessels on the optic disc (NVD), new vessels elsewhere (NVE), preretinal or vitreous haemorrhage, preretinal fibrosis and tractional retinal detachment
5. R3s = Retinal laser photocoagulation spots and stable retina during hospitalization or shortly after discharge

Digital retinal images were graded by both the community general practitioner and the expert ophthalmologist. DR scoring by an expert ophthalmologist was considered the gold standard. Patients diagnosed with R2 or R3a by ophthalmologist were referred to the ophthalmology department of Nanchang First Hospital for further examination and treatment. Taking the DR scoring by expert ophthalmologist as a control, the sensitivity, specificity and accuracy of the scoring by the community general practitioner were calculated.

### Statistical analysis

The diagnostic results of the two examiners were statistically analysed, and the diagnostic test was evaluated with the SPSS 25.0 software package. The consistency/kappa value was taken as the index. When the kappa value was less than 0.6, the consistency was poor. When the kappa value was between 0.6 and 0.75, the consistency was moderate. When the value exceeded 0.75, the consistency was high. The counting data, such as sensitivity, specificity and accuracy, were expressed by use cases (%), and the difference was statistically significant if *P* < 0.05 by the χ^2^ test.

## Results

### Overall patient characteristics.

We recorded data from 3511 eyes from a total of 1763 patients, of which 15 patients had only one eye. Among them, 326 eyes (9.29%) of 209 patients had poor fundus image quality and were not suitable for grading. Thus, only 3185 fundus images from 1646 patients were retained for assessment by the community general practitioner and ophthalmologist. The patients suitable for DR screening (n = 1646) had a median age of 58.64 ± 10.88 years, and 58.33% were male; the incidence rate of hypertension was 43.16%, and all the participants were of Chinese origin.

#### Analysis of the efficacy of community general practitioner in screening for diabetic retinopathy in 2020

A total of 1131 fundus images were retained for assessment. The ophthalmologist detected DR in 793 eyes, with 689 graded as R1, 54 as R2, 35 as R3a, and 15 as R3s. Community general practitioner detected DR in 673 eyes, with 533 graded as R1, 77 as R2, 44 as R3a, and 19 as R3s.The ophthalmologist detected DR in 70.11% (793/1131) of the fundus images and community general practitioner detected DR in 59.50% (673/1131) of the fundus images.The Kappa value was 0.578, and the examination results of ophthalmologist and community general practitioner were statistically significant through the consistency test. The consistency in the grading between the two doctors was poor (Table 1).

**Table 1:** The screening of diabetic retinopathy by different doctors in 2020.

Ophthalmologist	Community general practitioner					Total
	R0	R1	R2	R3a	R3s	
R0	304	34	0	0	0	338
R1	154	492	39	4	0	689
R2	0	7	25	14	8	54
R3a	0	0	10	22	3	35
R3s	0	0	3	4	8	15
Total	458	533	77	44	19	1131
Kappa	0.578					
P	0.000					

Table 2 describes the performance of community general practitioner and ophthalmologist in screening for DR in 2020. The sensitivity of community general practitioner in evaluating DR was 80.58% (639/793), the specificity was 89.94% (304/338), and the accuracy was 83.38% (943/1131).

**Table 2:** Sensitivity, specificity and accuracy of community general practitioner in the diagnosis of diabetes retinopathy in 2020.

Ophthalmologist	Community general practitioner		Sensitivity%	Specificity%	Accuracy%
	Positive	Negative			
Positive	639	154	80.58% (639/793)	89.94% (304/338)	83.38% (943/1131)
Negative	34	304			

#### Analysis of the efficacy of community general practitioner in screening for diabetic retinopathy in 2021

A total of 1452 fundus images were retained for assessment. The ophthalmologist detected DR in 1051 eyes, with 958 grade as R1, 58 as R2, 29 as R3a, and 6 as R3s. Community general practitioner detected DR in 1089 eyes, with 963 graded as R1, 88 as R2, 31 as R3a, and 7 as R3s.The ophthalmologist detected DR in 72.38% (1051/1452) of the fundus images and community general practitioner detected DR in 75.00% (1089/1452) of the fundus images.The Kappa value was 0.685, which was better than the previous year, and it was statistically significant through the consistency test (Table 3). The consistency in grading between the two doctors was moderate.

**Table 3:** The screening of diabetic retinopathy by different doctors in 2021.

Ophthalmologist	Community general practitioner					Total
	R0	R1	R2	R3a	R3s	
R0	315	85	1	0	0	401
R1	48	864	46	0	0	958
R2	0	14	30	10	4	58
R3a	0	0	9	18	2	29
R3s	0	0	2	3	1	6
Total	363	963	88	31	7	1452
Kappa	0.685					
P	0.000					

Table 4 describes the performance of community general practitioner and ophthalmologist in screening for DR in 2021. The sensitivity of community general practitioner in evaluating DR was 95.43% (1003/1051), the specificity was 78.55% (315/401), and the accuracy was 90.77% (1318/1452).

**Table 4:** Sensitivity, specificity and accuracy of community general practitioner in the diagnosis of diabetes retinopathy in 2021.

Ophthalmologist	Community general practitioner		Sensitivity%	Specificity%	Accuracy%
	Positive	Negative			
Positive	1003	48	95.43% (1003/1051)	78.55% (315/401)	90.77% (1318/1452)
Negative	86	315			

#### Analysis of the efficacy of community general practitioner in screening for diabetic retinopathy in 2022

A total of 602 fundus images were assessed. Ophthalmologist detected DR in 466 eyes, with 426 graded as R1, 24 as R2, 14 as R3a, and 2 as R3s. Community general practitioner detected DR in 453 eyes, with 398 graded as R1, 37 as R2, 14 as R3a, and 4 as R3s. The ophthalmologist detected DR in 77.41% (466/602) of the fundus images and community general practitioner detected DR in 75.25% (453/602) of the fundus images.The Kappa value was 0.744, which was the best during the three years, and it was statistically significant through the consistency test (Table 5).

**Table 5:** The screening of diabetic retinopathy by different doctors in 2022.

Ophthalmologist	Community general practitioner					Total
	R0	R1	R2	R3a	R3s	
R0	121	15	0	0	0	136
R1	28	379	19	0	0	426
R2	0	4	17	2	1	24
R3a	0	0	1	11	2	14
R3s	0	0	0	1	1	2
Total	149	398	37	14	4	602
Kappa	0.744					
P	0.000					

Table 6 describes the performance of community general practitioner and ophthalmologist in screening for DR in 2022. The sensitivity of community general practitioner in evaluating DR was 93.99% (438/466), the specificity was 88.97% (121/136), and the accuracy was 92.86% (559/602).

**Table 6:** Sensitivity, specificity and accuracy of community general practitioner in the diagnosis of diabetes retinopathy in 2022.

Ophthalmologist	Community general practitioner		Sensitivity%	Specificity%	Accuracy%
	Positive	Negative			
Positive	438	28	93.99% (438/466)	88.97% (121/136)	92.86% (559/602)
Negative	15	121			

The accuracy of community general practitioner in diagnosing DR increased yearly during the three years (*P* < 0.05), as shown in Table 7.

**Table 7:** Sensitivity, specificity and accuracy of community general practitioner in the diagnosis of diabetes retinopathy in these three years.

Time	Cases	Sensitivity%	Specificity%	Accuracy%
2020	1131	80.58% (639/793)	89.94% (304/338)	83.38% (943/1131)
2021	1452	95.43% (1003/1051)	78.55% (315/401)	90.77% (1318/1452)
2022	602	93.99% (438/466)	88.97% (121/136)	92.86% (559/602)
Total	3185	90.04% (2080/2310)	84.57% (740/875)	88.54% (2820/3185)
χ^2^		121.37	20.62	47.89
P		0.000	0.000	0.000

## Discussion

In recent years, with the increasing incidence rate of diabetes, the number of patients with blindness caused by DR has increased yearly, posing a serious threat to their health and quality of life^4^. One of the common complications of diabetes is DR. DR easily leads to serious complications of vision loss in patients with this disease. In this study, the ophthalmologist detected DR in 72.53% ((793 + 1051 + 466)/(1131 + 1452 + 602)) of the fundus images, which was higher than that in other studies^5,6^. This may be due to the following two reasons. There may have been sampling bias in our study, as our patients came from the largest hospital in Nanchang city, where severe cases are usually treated. The people we enrolled were different and were inpatients during the COVID-19 pandemic, which means that they may have had more complications; otherwise, they may not have gone out to see a doctor. Second, the diagnostic criteria were also different.

Seven-field fundus retinography was previously considered the gold standard for DR screening, but obtaining these images may be time-consuming and expensive, limiting their use^7^. The results reported by Fernando and colleagues showed that analysing a single fundus image per eye for DR detection achieved satisfactory accuracy^8^. Some authors support the use of dilated pupils to improve diagnostic performance^9,10^, but given the risk of serious complications such as acute angle-closure glaucoma, especially in the absence of an ophthalmologist, the improvement that dilated pupils may provide may not be worthwhile. In the study by Siu et al., ophthalmologists found that nonmydriatic fundus retinography was more sensitive than direct ophthalmoscopy (64% vs. 41%) in detecting DR in diabetes patients^7^. Therefore, only one 45° colour photo of the posterior pole of the fundus was taken with the macula as the centre by a nondilated fundus camera in this study.

Research reports from other countries have shown that nonmedical graders and assistant health care professionals are very sensitive in the evaluation and grading of DR^11,12^. The sample size of this study was greater. Limited evidence on the reliability of community general practitioners' grading of DR from digital retinal photographs is available^13–15^. In this study, we found that community general practitioner had satisfactory sensitivity (90.04%), specificity (84.57%) and accuracy (88.54%) compared with expert ophthalmologist through teleophthalmology consultations. Moreover, accuracy continuously improved through training on the website and long-term practice. Rosses et al. found that trained family doctors had high sensitivity (82.9%), specificity (92%) and accuracy (90.3%) in evaluating DR in diabetes patients, and the consistency with ophthalmologists (kappa adjustment coefficient: 0.74–0.80) was excellent^10^. Romero et al. used a nonmydriatic fundus camera to evaluate 779 patients with diabetes and found that there was substantial consistency between family doctors and ophthalmologists (kappa coefficient: 0.82), with high sensitivity (95.2%) and specificity (98%) for DR diagnoses^16^. The inclusion of nonophthalmic medical personnel in DR screening programs is feasible. This study demonstrated that cooperation between ophthalmologists and nonophthalmic medical personnel in DR screening is necessary, and it is possible to solve the current shortage of eye care personnel.

The community general practitioner was able to grade photos in the R0 category as high as 84.57%(740/875) correctly after gaining skills from the website. However, there was an unsatisfactory proportion of misclassification in the case of the R2, R3a and R3s categories, especially R2. The community general practitioner mistakenly graded some photos as R1 or R3a and R3s, with an error rate of nearly 50% ((7 + 14 + 8 + 14 + 10 + 4 + 4 + 2 + 1)/(54 + 58 + 24)), and more of them were misdiagnosed as R3a and R3s. This is different from the research results of Islam^17^ and may be related to the longer training time in Islam's study. Since the R1 category requires participants to undergo a second retinal examination after half a year, R3s means retinal laser photocoagulation spots and stable retina during hospitalization or shortly after discharge, and the R2 and R3a categories require immediate referral to an ophthalmologist, this error may not be too serious at the screening stage. However, the high misdiagnosis and missed diagnosis rates suggest that continuous training of community general practitioners is very important. Fortunately, as their experience increases, their accuracy is expected to improve.

The strengths of this study are as follows: The diagnostic accuracy of community general practitioner has continuously improved to a satisfactory level, which enables patients to obtain DR screening reports in real time during the same hospital visit so that the necessity of referral and treatment can be directly explained to patients by community general practitioners. Second, the sample size was large. In view of the advantages of this system, it is recommended to perform DR screening during the follow-up of diabetes patients to better screen these patients. This is an excellent model that can be followed and easily expanded.

This study also had several limitations. In this study, only one 45° colour photo of the posterior pole of the fundus was taken with the macula as the centre, which means that some of the peripheral retinal lesions may have been missed, and the DR grade may have been underestimated^18^. Second, the consistency of the community general practitioner's diagnosis still needed to be improved; otherwise, the risk of misdiagnosis or medical errors may increase, which needs to be further explored and improved. Finally, this study excluded those who had a seriously affected quality of photos due to unclear refractive media. Most of the patients were over 70 years old, possibly with cataracts or corneal opacity. This is indeed a limitation of remote screening. For those patients, it is recommended that they go to a superior hospital for ophthalmological examination as soon as possible.

The purpose of this study was to help more people conduct regular screening and compensate for the shortage of trained ophthalmologists required for DR screening in terms of time, distance and availability. This study provided evidence that remote ophthalmology should be expanded and included in the follow-up model of diabetes in all parts of China so that any institution treating diabetes and comorbidities (even community hospitals) can quickly cover DR screening. Teleophthalmology screens the fundus of diabetes patients online, overcomes the outdated conventional guidance, reduces the number of times patients have to visit the hospital, avoids the risk of cross contamination of COVID-19, enables diabetes patients to receive accurate guidance during the COVID-19 pandemic, and lightens the economic burden of families and the country. At the same time, ophthalmologists can see patients' fundus images through computers or even smartphones, which overcomes the limitations of doctors' workspaces.

## Data Availability

The datasets generated and/or analysed during the current study are not publicly available due [REASON WHY DATA ARE NOT PUBLIC] but are available from the corresponding author upon reasonable request.
